# Editorial: Understanding obesity to determine the best therapeutic option: from lifestyle interventions to therapies

**DOI:** 10.3389/fnut.2025.1560942

**Published:** 2025-02-11

**Authors:** Evelyn Frias-Toral, Florencia Ceriani, Jorge Carriel-Mancilla, Almino Ramos

**Affiliations:** ^1^School of Medicine, Universidad Católica de Santiago de Guayaquil, Guayaquil, Ecuador; ^2^Nutrition School, Universidad de la República (UdelaR), Montevideo, Uruguay; ^3^GastroObesoCenter, Institute for Metabolic Optimization, São Paulo, Brazil

**Keywords:** obesity, therapeutic intervention, lifestyle change, gut microbiota, pharmacological therapies

Obesity is a global health crisis that transcends age, geography, and socioeconomic barriers, significantly increasing the risk of type 2 diabetes, cardiovascular diseases, cancer, and other chronic conditions. The urgency to tackle this multifaceted epidemic requires a multidisciplinary approach that integrates molecular, behavioral, and clinical perspectives. This Research Topic, “*Understanding Obesity to Determine the Best Therapeutic Option*,” presents pivotal research aimed at advancing precision medicine and patient-centered approaches for obesity management.

A cornerstone for addressing obesity lies in the promotion of healthy lifestyle habits. Recent findings underscore the importance of discipline-specific interventions to promote health equity across diverse populations (1). Dietary strategies remain central in obesity research. Various types of diets, including low-calorie, low-carbohydrate, and high-protein regimens, are tailored to individual needs. Studies highlight the efficacy of the Mediterranean diet in promoting weight loss and improving metabolic health, due to its rich nutrient profile and anti-inflammatory properties (2). Additionally, ketogenic diets have shown promise by enhancing fat oxidation and reducing appetite, making them viable options for obesity management (3).

Pharmacological therapies are progressively recognized as effective alternatives for obesity treatment, offering promising results for individuals who struggle with lifestyle-based interventions. GLP-1 receptor agonists, such as semaglutide and liraglutide, have demonstrated significant weight loss and metabolic improvements (4) (Tamayo-Trujillo et al.). Moreover, combination therapies targeting appetite regulation and energy expenditure are emerging as innovative approaches in obesity management (Vedrenne-Gutiérrez et al.). Bariatric surgery is another highly effective intervention for managing severe obesity, leading to substantial weight loss and improvements in comorbidities. Studies have shown these procedures are associated with a 59% reduction in all-cause mortality among obese adults with type 2 diabetes and a 30% reduction among those without that condition (5).

Emerging research highlights the roles of genetic and microbial determinants in obesity, providing insights for personalized treatments. Genome-wide association studies (GWAS) have identified genetic variants linked to obesity susceptibility, such as those in the FTO gene, which influence appetite and energy regulation (6). Concurrently, studies reveal that gut microbiota composition significantly affects energy balance and metabolic health, with interventions targeting microbiota showing potential for obesity management (7).

Environmental factors also play a crucial role in obesity risk (8, 9). A 2024 scoping review identified endocrine-disrupting chemicals (EDCs), such as bisphenols, phthalates, parabens, and triclosan, as key contributors, emphasizing the need for preventive strategies (10). These findings stress the importance of considering environmental influences when designing obesity prevention strategies.

Collectively, the 15 manuscripts in this Research Topic offer a comprehensive exploration of the mechanisms and interventions for obesity, spanning lifestyle changes to advanced therapies. These findings highlight the importance of a multidisciplinary and personalized approach to effectively combat this complex public health challenge (Reytor-González et al.).

Lifestyle interventions continue to be indispensable. Studies exploring the Health at Every Size (HAES) paradigm advocate for sustainable and body-positive approaches (Suárez et al.) Meanwhile, innovative dietary strategies, such as intermittent fasting, reveal promising effects on gut microbiota composition and metabolic health, warranting further exploration of their long-term impacts (Cadena-Ullauri et al.). Furthermore, cashew nut and oil interventions demonstrate the potential of dietary bioactives to improve cardiometabolic risk profiles during weight loss (Meneguelli et al.).

Emerging research underscores the critical role of body composition in obesity-related risks. For example, oxidative balance scores provide insights into the interplay between dietary antioxidants, prooxidants, and obesity, offering new avenues for targeted interventions (Zhu et al.). Suárez et al. reported that assessing muscle mass can help detect adult individuals with high risk of developing type 2 diabetes as one of the obesity-related comorbidities. It is essential to promote healthier eating and lifestyle habits among young individuals, as highlighted by Saintila et al., who reported insufficient consumption of whole grains, legumes, vegetables, nuts, and seeds, along with inadequate levels of regular physical activity, hydration, and sunlight exposure among Peruvian university students.

Notably, as reported by Arteaga-Pazmiño et al., understanding dynapenic obesity, the coexistence of excess body weight and low muscle strength, has proven essential for addressing physical performance impairments in aging populations. Additionally, the influence of skeletal muscle weight on anesthesia dosing in obese patients exemplifies the importance of integrating body composition analysis into clinical practice (Hu et al.).

Micronutrient status also plays a vital role in obesity management. Studies in Research Topic highlight the importance of tailored supplementation to address common deficiencies in vitamin D, calcium, and iron (Côté et al.). Interestingly, Zambrano et al. point out that understanding the changes in gut microbiota following bariatric surgery is essential for predicting metabolic outcomes and developing targeted interventions to optimize obesity management. Furthermore, when developing nutrition programs for post-bariatric surgery patients, it is essential to involve a specialist. This approach ensures that frequently reported macro- and micronutrient deficiencies, as highlighted by the study from Afsar and Ozdogan, are effectively addressed before initiating supplementation.

Also, it could be mentioned the broader societal implications of obesity. For instance, Díaz et al. reported the links between obesity and hypovitaminosis D revealing how socio-environmental factors interact with physiological mechanisms.

In conclusion, the multifaceted nature of obesity requires a multidisciplinary and personalized approach to its management. This Research Topic emphasizes the critical role of lifestyle interventions, innovative dietary strategies, and pharmacological advancements in combating obesity. Emerging research on genetic predispositions, gut microbiota, and body composition provides insights into tailored interventions for prevention and treatment. Furthermore, addressing micronutrient deficiencies, particularly post-bariatric surgery, highlights the importance of specialized nutritional strategies. By integrating environmental and socio-cultural factors, these findings pave the way for holistic strategies to effectively tackle this global health crisis.

## References

[1] Alkhatib A Barengo NC. Editorial: Physical activity, health equity and health-related outcomes, volume II. Front Public Health. (2024) 12:1379960. 10.3389/fpubh.2024.1379960 38435284 PMC10905956

[2] Muscogiuri G Verde L Sulu C Katsiki N Hassapidou M Frias-Toral E. Mediterranean diet and obesity-related disorders: what is the evidence? Curr Obes Rep. (2022) 11:287–304. 10.1007/s13679-022-00481-1 36178601 PMC9729142

[3] Barrea L Caprio M Grassi D Cicero AFG Bagnato C Paolini B. A new nomenclature for the very low-calorie ketogenic diet (VLCKD): very low-energy ketogenic therapy (VLEKT) Ketodiets and nutraceuticals expert panels: “KetoNut”, Italian Society of Nutraceuticals (SINut) and the Italian Association of Dietetics and Clinical Nutrition (ADI). Curr Nutr Rep. (2024) 13:552–6. 10.1007/s13668-024-00560-w 39039372 PMC11327192

[4] Moore PW Malone K VanValkenburg D Rando LL Williams BC Matejowsky HG. GLP-1 Agonists for weight loss: pharmacology and clinical implications. Adv Ther. (2023) 40:723–42. 10.1007/s12325-022-02394-w 36566341

[5] Syn NL Cummings DE Wang LZ Lin DJ Zhao JJ Loh M. Association of metabolic-bariatric surgery with long-term survival in adults with and without diabetes: a one-stage meta-analysis of matched cohort and prospective controlled studies with 174 772 participants. Lancet. (2021) 397:1830–41. 10.1016/S0140-6736(21)00591-2 33965067

[6] Loos RJF Yeo GSH. The genetics of obesity: from discovery to biology. Nat Rev Genet. (2022) 23:120–33. 10.1038/s41576-021-00414-z 34556834 PMC8459824

[7] Sarmiento-Andrade Y Suárez R Quintero B Garrochamba K Chapela SP. Gut microbiota and obesity: new insights. Front Nutr. (2022) 9:1018212. 10.3389/fnut.2022.1018212 36313072 PMC9614660

[8] Muscogiuri G Barrea L Frias-Toral E Garcia-Velasquez E de Angelis C Ordoñez C. Environmental impact on metabolism. In:Pivonello R Diamanti-Kandarakis E, editors. Environmental Endocrinology and Endocrine Disruptors. Endocrinology. Cham: Springer (2023), 397–42. 10.1007/978-3-030-39044-0_14

[9] Verde L Frias-Toral E Cardenas D. Editorial: Environmental factors implicated in obesity. Front Nutr. (2023) 10:1171507. 10.3389/fnut.2023.1171507 37215212 PMC10192899

[10] Amon M Kek T Klun IV. Endocrine disrupting chemicals and obesity prevention: scoping review. J Health Popul Nutr. (2024) 43:138. 10.1186/s41043-024-00627-y 39227884 PMC11373446

